# The Effect of Baby Food E-Store Image (for Ages 0–3) on Consumers’ Purchase Intention

**DOI:** 10.3389/fpsyg.2021.796750

**Published:** 2021-11-30

**Authors:** Chunlan Jiao, Xiangdong Shen, Li Wang

**Affiliations:** ^1^Normal School, Changshu Institute of Technology, Changshu, China; ^2^Business School, Changshu Institute of Technology, Changshu, China; ^3^School of Business Administration, Anhui University of Finance & Economics, Bengbu, China

**Keywords:** S-O-R framework, baby food e-store image, perceived value, purchase intention, mediating effect

## Abstract

This research aims to investigate the influence of baby food e-store image (for ages 0–3) on consumers’ purchase intention, mainly based on the stimulus-organism-response (S-O-R) model. Perceived value is additionally tested as a mediator of the relationship between baby food e-store image and consumers’ purchase intention. An online questionnaire survey was conducted among consumers of baby food e-stores that specialize in ages 0–3. The final sample comprised 584 valid responses. To test the hypotheses in the relationships among latent variables, structural equation model analysis was used in this study. The findings showed that design image, order fulfillment image, communication service image, and security image of baby food e-stores have positive effects on consumers’ perceived value, while perceived value mediates the relationship between baby food e-stores and consumers’ purchase intention. As a result, more perceived value would be created between online stores and their consumers with a higher level of value. Two managerial implications derived from this study explain how to manage baby food e-stores and how to promote online baby food undertakings. Particularly, consumers’ perceived emotional and functional value can be shaped by four dimensions of baby food e-store image, e-store design image, order fulfillment image, communication service image, and security image, which finally determines their purchase intentions. The originality and value of this study enriches the importance of consumers’ perceived value in the field of online store image. Moreover, this study demonstrates that baby food e-store image increases consumers’ perceived value and purchase intention.

## Introduction

China’s three-child policy has accelerated the arrival of the “baby boom” across the country, and the rapidly growing number of newborn babies in China has introduced a stable target consumer group in the maternal and baby markets. According to the monitoring data of the annual comprehensive analysis of China’s Internet Maternal and Child Market 2019, the number of births in 2016 rose to 17.86 million, of which the second child accounted for more than 50% of these births. Since then, favorable policies for childbirth have been introduced continuously (National Bureau of Statistics, 2021). The increase in the number of newborn babies and the increase in the proportion of second child and third childbirths indicate that the maternal and baby products market will continue to expand in the next few years. Consequently, that both physical store sales and online platform sales will likely show a rapid growth trend (Xinhua News Agency, 2021). Monitoring data from Analysys Enfodesk, a major market tracking company, shows that the retail scale of maternal and baby online retail in 2019 reached $ 77.6 billion (Analysys Enfodesk, 2020).

Many businesses have seen potential opportunities in the online baby product market and have settled on e-commerce platforms to establish online baby food stores. With the development of smartphones and 4G/5G networks, online shopping for maternal and baby products has infiltrated the lives of the general public. New concepts of nurturing and consumption upgrades have caused consumers to have higher requirements for baby food products. Furthermore, online shopping channels for baby food products are diversified. Therefore, in the face of the ever-changing industry structure and the fierce competition in the e-commerce environment, online baby food store operators must conform to development trends of the e-commerce era and effectively use Internet tools to enhance the image of the online store and increase customer stickiness. Through the establishment of high-quality and stable customer relationships, profitability and sustainable development can be achieved.

One of the purposes of this study is to expand the baby food e-store image and ensuing consumer behaviors in the context of the Internet. Hence, it is proposed that perceived emotional value and perceived functional value mediate the relationship between baby food e-store image and purchase intention. In addition, to develop the framework of stimulus-organism-response (S-O-R), a more comprehensive and rigorous understanding of the influencing factors of consumers’ by the baby food e-store image is needed. The findings of this study are expected to extend the S-O-R framework, perceived value and provide practical implications for the development of the baby food e-store image and strategies for the “ages 0–3” market segment.

This study contributes to the literature in several ways. First, by examining the existing research on online store images, it was found that most literature focuses on adults, and there was little research on babies (ages 0–3 years old). This study focuses on the baby food e-store image (for ages 0–3) in online stores selling baby food and expands the research scope of online store image. Second, most of the previous studies in the field of online store image do not consider the different dimensions of online stores. This study divides the baby food e-store image into four dimensions, enriching the research dimension of baby food e-store image. Third, this study explores multiple mediating effects of the two dimensions of perceived value, which enriches the research on perceived value. This is an important application of structural equation model (SEM) research methods. Fourth, this study applies the S-O-R model to the study of the baby food e-store image, which is an expansion of the application field of the S-O-R framework.

This study consists of five parts. The theoretical background of this study follows the introduction and is presented in the second section. The third section encompasses the research framework and hypotheses development. Research methods and data analysis are analyzed in detail in the fourth section, and the final section discusses the findings and highlights of the theoretical and practical implications for researchers and companies, as well as suggestions for future research based on the limitations of this study.

## Theoretical Background

### The Stimulus-Organism-Response Framework

An S-O-R paradigm is applied for the framework building of this research, and there are three reasons for choosing this paradigm. Firstly, though the S-O-R paradigm was initially used in the field of online psychology, marketing researches also demonstrated that consumers’ consumption behaviors could be influenced by internet stimuli, such as store atmospheric cues, social culture (Eroglu et al., 2003; Mazaheri et al., 2011). Especially for the Chinese people who are collectivism dominated in the multicultural social environment would be more sensitive to various environmental cues (Shobeiri et al., 2018). Thus, the S-O-R paradigm could be adapted in this research to investigate the China’s consumers’ responses to external stimulus. Secondly, within the S-O-R paradigm, consumers’ internal state, such as emotion, also have effect on their approaches or avoidance actions when they accept the multisensory input from external environment as well as stimuli input from online retailers (Eroglu et al., 2003). Some researches use S-O-R paradigm to explain consumers’ consumption behaviors stimulated by various external environments, and some predictions have been made by using S-O-R paradigm (Russell and Mehrabian, 1974; Wu et al., 2020). Therefore, S-O-R model could be used to explain the relationship among infants food e-store image of aged 0–3, perceived values and consumers’ purchase intention. The research carried out by Shobeiri et al. (2018) also experimentally provided evidences showing that consumers’ internal process could play the mediation role in the relationship between external environment stimuli and consumers’ consumption behavior. So, one aim of this study is exploring how the perceived value as the mediation variable has put the effect in the relationship between the infants food e-store image of aged 0–3 and consumers’ purchase intention. Thirdly, considering the continuous expansion and deepening of China’s internet industry, related studies which focus on investigating into which features could capture consumers’ true intentions, satisfy their inner needs and further increase their online consumer behaviors, have shown high research importance and practical significance within the Chinese online marketing management framework (Eroglu et al., 2003; Chai et al., 2017; Li et al., 2020). So it is appropriate to apply the S-O-R paradigm to this study for examining the relationship between the infants food e-store image of aged 0–3 and consumers’ purchase intention. To sum up, the S-O-R paradigm is an effective and straightforward method for this study to discuss the consumers’ responds to internet behaviors by examining the perceived value as a mediator on the relationship between infant food e-store image of aged 0–3 as multisensory stimulus and consumers’ purchase intention.

### Baby Food E-Store Image (for Ages 0–3)(S)

In recent years, the online commerce market has developed rapidly, and the image of online stores has attracted the attention of consumers and academia. Research on the image of online stores will become an important supplement to the theory of store image. Aladwani et al. (2002) developed four indicators to judge consumers’ perceptions and perceptions of online stores, including online store technology, design image, store content, and quality. Ranganathan and Ganapathy (2002) summarized the characteristics of retail online stores, and concluded that the dimensions of online store image include five dimensions: design, information content, services, goods, and security. Helmefalk (2016) summarized the traditional store image and pointed out that the dimensions of online store image can be divided into seven dimensions: familiarity, ease of use, online store type, pleasure, usefulness, trust, and settlement ability; Heijden and Verhagen (2004) also divided the online store image into seven dimensions according to the psychological attributes of the store image, which included ease of use, the usefulness of the store, style, pleasure of shopping, trust, online store solutions, and familiarity with online stores. Jin and Park (2006) divided the online store image into six dimensions, including web design image, communication image, order fulfillment image, product image, security image, and promotion image based on the functional attributes of the online store. In consideration of the current online environment in China and the characteristics of integrated B2C online stores, and to reflect consumers’ perception of the functionality of online stores, this study shall refer to the research results of Jin and Park (2006), by considering the current baby food e-store image and dividing the image into four dimensions. These dimensions include the image of the e-store design, the image of order fulfillment, the image of communication services, and the image of security and confidentiality.

### Perceived Value (O)

From the perspective of consumer psychology, Zeithaml (1988) pointed out that perceived value is the comparison between the customer’s benefits and the cost paid during the buying and selling process. Monroe (2012, p.145) believed that perceived value is “the ratio between perceived gains and perceived losses.” He maintained that consumers’ perception of value is manifested as a comparison between perceived product quality or perceived benefits and perceived costs; based on previous studies, this study combines the characteristics of China’s online baby food stores and maintains that perceived value is not only the comparison of the income and the cost but also the consumers’ expectations and feelings for a specific product or service.

In the existing literature, it is relatively common to use a single-dimensional, single-item scale to measure consumers’ perceived value. However, many scholars believe that perceived value is quite complex, and a single item measurement method cannot adequately meet research needs. Sheth et al. (1991) proposed five factors that affect perceived value, including emotional value, functional value, social value, conditional value and epistemic value. Yan (2019) divided the perceived value dimensions into six dimensions: social value, cognitive value, price and quality, satisfaction, image value, and emotional value. A scale of 33 items was used to measure the perceived value of educational services. However, Wang et al. (2019) believed that potential risks in the process of purchasing products or services should also belong to the dimension of customer perceived value and that it is a third dimension independent of perceived quality and price. Hallem and Barth (2011), based on the research of Sheth et al. (1991), divided consumers’ perceived value into two dimensions: emotional value, and functional value. Emotional value refers to the utility that customers perceive when purchasing goods or services. Functional value includes two aspects: price and quality. Price refers to the rational price paid by consumers for purchasing goods or services, and quality refers to the utility of the perceived quality and functionality of the product. This study focuses on the dimensions and maturity scales of Hallem and Barth (2011), combined with the views of domestic experts and scholars, to measure the perceived value of consumers and conduct an in-depth study of the perceived value of online consumers in China.

## Research Model and Hypotheses

### Conceptual Framework

**FIGURE 1:**
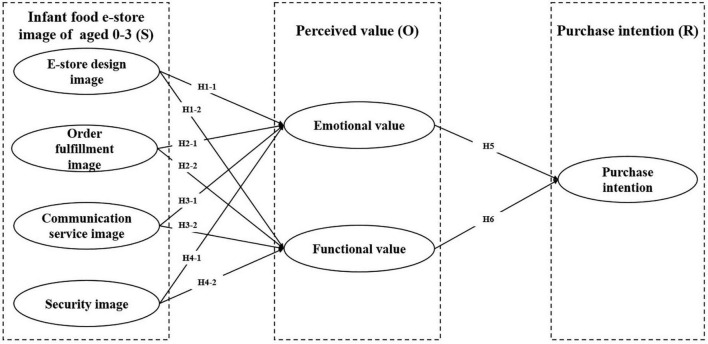
Conceptual framework.

Based on the theoretical basis of this research, this research based on S-O-R paradigm, combines infants food e-store image of aged 0–3, perceived value, and purchase intention establishes the research model of this research, as shown in Figure 1.

### Research Hypotheses

#### The Relationship Between E-Store Design Image and Perceived Value

Online shop web design is the design of the art and functions involved in the online shop, which mainly includes the pages’ art design, layout and level, color and style, and search function, as well as advertising space, membership services, and other content. Web page design should conform to the habits of online consumers and reflect convenience and selectivity. Page and program design should have practical and convenient functions, and the overall tone and layout should appeal to consumers (Yun and Linda, 2007). Other considerations could include features such as detailed product descriptions, a sharing and communication platform for consumers, convenience, accurate search functions to help consumers quickly find the products they need. Chang and Chen (2008) pointed out that most of the failed online stores are a result of the online store design deviating from the habits of online consumers, causing consumer rejection. The landing page of an online store is the first step in the consumers’ experience and is a prerequisite for consumers to decide whether to continue using the online store; therefore, web design is an important factor in determining whether consumers will continue to browse the online store. Exquisite webpage design has an impact on consumers’ vision. Pleasing webpage design helps consumers feel comfortable and richly functional webpage design helps consumers enjoy the process, which will generate the desire to continue browsing and searching the online store, thus enhancing the emotional value for consumers. By contrast, poorly designed and poorly functional webpages hinder consumers’ desires and buying interest. Therefore, this study proposes the following hypotheses:

H1-1: E-store design image has a significant positive impact on emotional value.
H1-2: E-store design image has a significant positive impact on functional value.

#### The Relationship Between Order Fulfillment Image and Perceived Value

Beth et al. (2011) emphasized order fulfillment should be a consistent quality of e-commerce platform websites to fulfill their promise to customers. Shu and Yang (2011) indicated that online stores that arrange delivery for customers at a fixed time or at a promised time reduce consumers’ perceived risks; therefore, consumers can save a lot of time and energy without worrying about whether the seller will deliver the goods on time. Furthermore, Hu et al. (2008) pointed out that if the promises announced by e-commerce platform companies on the website can be implemented well, their image will have a positive impact on consumers, thereby enhancing consumers’ perception of the functional value of online stores. If the promises of online stores cannot be fulfilled well, consumers will feel deceived by dishonest and inconsistent promises, which will weaken consumers’ perception of functional value. When the online store’s behavior is consistent with or exceeds its promise, consumers will love and connect with this type of online store more emotionally. Emotional value will be enhanced by the improvement of the online store’s order-fulfillment image. On the contrary, if the image of order fulfillment is poor, the emotional value of consumers will be weakened, causing consumer resistance, which in turn will affect their purchase behavior in online stores. Therefore, this study proposes the following hypotheses:

H2-1: Order fulfillment image has a significant positive impact on emotional value.
H2-2: Order fulfillment image has a significant positive impact on functional value.

#### The Relationship Between Communication Service Image and Perceived Value

Unlike physical stores, consumers and online store sales staff cannot directly communicate face-to-face but can send text narratives and pictures through chat software. This kind of information transmission method can be easily distorted by external noises, resulting in deviations in understanding. Therefore, the image of the communication service should include the service attitude of the customer service staff of the online store, ensuring questions are answered timely and professionally, consumer suggestions can be accepted, and consumers can accurately be provided with the information they need. Alkalbani and Hussain (2021) pointed out that trust is generated through frequent communication and exchange between buyers and sellers. In an online shopping environment, consumers cannot communicate frequently with online stores or experience face-to-face communication; therefore, it is difficult to establish trust in the online environment. Therefore, online shop customer service staff should focus on the consumer’s point of view and receive online consumer communications in a polite, honest, patient, and enthusiastic manner, so that consumers feel respected, valued, and satisfied. This will produce a pleasant shopping experience, and as a result, consumers’ emotional value will increase. Therefore, this study proposes the following hypotheses:

H3-1: Communication service image has a significant positive impact on emotional value.
H3-2: Communication service image has a significant positive impact on functional value.

#### The Relationship Between Security Image and Perceived Value

Kutty (2020) maintained that security and confidentiality are necessary measures for e-commerce platforms to protect consumer privacy information and online transactions. Eriksson et al. (2021) pointed out that consumers who purchase goods online do not have personal contact with the online store or physical objects, so they feel the existence of transaction risks. Consumers are mainly worried about the following aspects: whether personal information is easily leaked, whether the payment method for online transactions is safe, whether there is a risk of stolen funds, whether the online store has clear commitments and compensation measures, and whether the online store has adopted advanced technology to improve shopping safety. Patil and Sudhir (2020) suggested that the image of security and confidentiality is the basis for easy shopping and an important guarantee for consumers to make safe online consumption. Therefore, this study proposes the following hypotheses:

H4-1: Security image has a significant positive impact on emotional value.
H4-2: Security image has a significant positive impact on functional value.

#### The Relationship Between Perceived Value and Purchase Intention

From the perspective of consumers’ psychology discovered through many empirical studies, Zeithaml (1988) concluded that during the shopping process, the greater the benefits consumers perceive about products or services, the higher their perceived value, which in turn promotes their willingness to purchase goods or services. Peng et al. (2019) pointed out that the real driving factor of consumers’ purchasing behavior is perceived value. Chae et al. (2020) believes that the perceived value of the Internet is the comparison of online consumers’ benefits and payments. When consumers believe that the benefits of a product or service are greater than the benefits they have paid, the perceived value is positive, resulting in purchase willingness. When the perceived benefit is far greater than the cost, the willingness to buy will be stronger. From the perspective of online consumers, purchase intentions or behaviors are derived from their demand for goods or services. In the online shopping process, consumers will perceive the functional value of the desired goods or services, which will affect their willingness to buy in online stores. In addition, consumers also have certain preferences during the purchase process, which includes consumers’ emotional factors. In other words, under the same conditions, consumers choose their favorite products or services. Therefore, based on customer value theory, this study proposes the following hypotheses:

H5: The perceived value of emotional value has a significant positive impact on consumers’ purchase intentions.
H6: The perceived value of functional value has a significant positive impact on consumers’ purchase intentions.

#### The Mediating Effect of Perceived Value

An online store’s image is the psychological perception of consumers in the process of online shopping. Like perceived value, online consumers’ perception of an online store is also a subjective feeling at the psychological level, that is, whether they have a sense of identity with the online store’s image, whether they agree that the online store’s image has a certain value, and whether they are willing to accept the online store. Therefore, the image of an online store will resonate with online consumers’ perception of value, which in turn affects the perceived value. Consumers’ good perception and high overall evaluation of online stores are derived from a good online store image, which is followed by an increase in consumer perceived value (Han and Kwon, 2009). Florence et al. (2020) pointed out that what customers buy and consume is not products but value. Most consumer behaviors are driven by value, which is a key factor in determining customer purchasing behavior. Monroe (2012) suggested that consumers’ purchase intentions are based on their cognitive value. Consumers will compare the actual price of goods or services with their own quality during the shopping process and determine the purchase intention through the measurement of value. In summary, the image of online stores affects consumers’ perceived value, which in turn affects consumers’ willingness to buy. Therefore, this study proposes the following hypotheses:

H7: Emotional value plays a mediating role between online baby food store image and consumers’ purchase intention.
H8: Functional value plays a mediating role between online baby food store image and consumers’ purchase intention.

### Research Methodology

#### Instrument

To ensure content validity, the items used to measure the constructs were adapted from extensive literature and modified to fit the study context. Measurement items for online store design image, order fulfillment image, communication service image, and security image were adapted from Jin and Park (2006) and Ranganathan and Ganapathy (2002). Measurement items for the perceived value of emotional value and functional value were adapted from Wang et al. (2019). Purchase intention was measured using three items adapted from Zeithaml (1988).

As the original items were in English, we conducted a back-translation to ensure translation validity. First, a researcher whose native language was Chinese translated the source items from English into Chinese. Next, another researcher independently translated these items back into English. Subsequently, the two researchers compared the two English versions and jointly revised the first Chinese version of the items. We then invited a panel of experts in the Internet consumer behavior field to examine the face validity of the survey instrument. Based on their feedback, minor modifications were made to improve the comprehensiveness and user-friendliness of the measurement items. A pre-test of the survey instrument was conducted to validate the instrument conceptually. The final survey questionnaire is presented in Appendix A. All items were measured on a 7-point Likert scale, ranging from 1 (not agree at all) to 7 (absolutely agree).

#### Data Collection

To ensure the external validity of the research and to ensure the representativeness of the survey sample, the survey scope of this study is limited to online baby food stores (for ages 0–3) in China. Only users who had participatory experience with online baby food stores (for ages 0–3) were included in this survey. Therefore, the representativeness of the sample was ensured for the survey. A survey was conducted through an online crowdsourcing platform in China, which provides functions equivalent to Amazon Mechanical Turk. The online survey platform used in this study was the most representative of China, thus ensuring the sample was representative of the survey method. The survey was conducted between August 1, 2021, and August 31, 2021. A total of 650 responses were received over a 4-week period. A total of 584 responses were used for subsequent analyses after 66 incomplete and invalid responses were excluded. Data were analyzed for statistical tests of the measurement model and hypotheses using IBM SPSS Statistics 24.0 and IBM AMOS Graphics 24.0. AMOS Graphics is a visual statistical program especially used for structural equation modeling, path analysis, and confirmatory factor analysis (CFA). AMOS Graphics not only overcomes the limitations of large sample conditions but also provides the standard error of path analysis indirect effects, which is particularly useful in the use of mediation effects. Therefore, AMOS Graphics was selected to analyze the SEM. Table 1 summarizes the demographic characteristics of the participants. Participants were relatively balanced in gender distribution, and the majority (44.9%) of the participants were between 21 and 30 years of age. Most participants were professionals and civil servants (51.5%). Furthermore, 33.2% of participants use online baby food stores (for ages 0–3) several times a week.

**TABLE 1:** Demographics of survey respondents (*N* = 584).

Demographics	Category	Frequency	%
Gender	Male	278	47.6
	Female	306	52.4
Age	Below 20	42	7.2
	21–30	262	44.9
	31–40	178	30.5
	41–50	71	12.2
	50 or above	31	5.3
Occupation	Office worker	113	19.3
	Civil servant	142	24.3
	Professional (Professor, Doctor, Lawyer, etc.)	159	27.2
	Homemaker	77	13.2
	Other	93	15.9
Use frequency	Several times a day	97	16.6
	Once per day	91	15.6
	Several times per week	194	33.2
	Several times per month	167	28.6
	Basically not used	35	6.0

## Data Analyses and Results

### Reliability and Validity

Following the two-step approach recommended by Anderson and Gerbing (1988), we first examined the measurement model to verify the reliability and validity of the instrument and then assessed the structural model.

We performed both principal component factor analysis and CFA to assess the reliability and validity of the scales. The Kaiser–Meyer–Olkin (KMO) statistics for the sample were 0.815, indicating that the data were amenable to factor analysis (Kaiser, 1974). All indicators loaded on the expected factors and were higher than 0.6, while loadings on other factors for all indicators were lower than 0.4, suggesting good convergent and discriminant validity (Chin et al., 1997).

Construct reliability and validity were further examined using the CFA. As shown in Table 2, the Cronbach’s α and composite reliability (CR) values for each construct ranged from 0.809 to 0.946, both of which were above the suggested threshold of 0.7 (Straub et al., 2004) and exhibited a satisfactory level of reliability. For construct validity, both convergent and discriminant validity were examined. Convergent validity was confirmed by examining the average variance extracted (AVE) and indicator loadings. As shown in Table 2, all AVE values were higher than the recommended level of 0.5 (Fornell and Larcker, 1981). The standard loadings of all items were above the desired threshold of 0.7 and significant at 0.001. This indicates a good convergent validity (Chin et al., 1997).

**TABLE 2:** Results of confirmatory factor analysis (CFA).

Construct	Indicator	Standard loading^a^	Cronbach’sα	CR	AVE
E-store design image	EDI 1	0.791	0.873	0.873	0.697
	EDI 2	0.865			
	EDI 3	0.846			
Order fulfillment image	OFI 1	0.898	0.866	0.870	0.691
	OFI 2	0.832			
	OFI 3	0.757			
Communication service image	CSI 1	0.836	0.877	0.880	0.649
	CSI 2	0.853			
	CSI 3	0.692			
	CSI 4	0.830			
Security image	SEI 1	0.773	0.821	0.822	0.606
	SEI 2	0.829			
	SEI 3	0.731			
Emotional value	EMV 1	0.788	0.913	0.917	0.788
	EMV 2	0.946			
	EMV 3	0.921			
Functional value	FUV1	0.790	0.863	0.863	0.613
	FUV 2	0.814			
	FUV 3	0.790			
	FUV 4	0.735			
Purchase intention	PI 1	0.811	0.809	0.811	0.589
	PI 2	0.711			
	PI 3	0.777			

*χ^2^ = 2.531, CFI = 0.955, TLI = 0.946, NFI = 0.928, and RMSEA = 0.051. ^a^All standard loadings were significant at p < 0.001.*

After examining the measurement validity and reliability, the proposed hypotheses were tested using AMOS. After modifying the original model, the actual and recommended values of the model fit indices are listed in Table 3. The fit indices of the model were better than the recommended thresholds, demonstrating a good fit between the model and the data.

**TABLE 3:** Results of discriminant validity testing.

Construct	M	S.D.	1	2	3	4	5	6	8
E-store design image	4.193	1.943	***0.835***						
Order fulfillment image	4.147	1.977	0.258	***0.831***					
Communication service image	4.680	1.766	0.208	0.116	***0.806***				
Security image	4.133	1.517	0.114	0.203	0.272	***0.778***			
Emotional value	4.477	1.828	0.243	0.279	0.194	0.183	***0.888***		
Functional value	3.475	1.821	0.345	0.335	0.197	0.228	0.152	***0.783***	
Purchase intention	3.717	1.580	0.216	0.177	0.287	0.301	0.326	0.333	***0.767***

*Diagonal bold italics entries are square roots of AVE; all others are correlation coefficients. M, mean; SD, standard deviation.*

Discriminant validity was assessed by comparing the square root of AVE for each construct with the correlations between that construct and other constructs (Fornell and Larcker, 1981). Table 4 indicates that the square roots of the AVEs (diagonal elements) were larger than the inter-construct correlations depicted in the off-diagonal entries, suggesting adequate discriminant validity.

**TABLE 4:** Measures of the model fit.

Fit index	*X*^2^/df	RMSEA	GFI	CFI	NFI	TLI
Recommended range	<3.84a	<0.08b	>0.90a	>0.90a	>0.90a	>0.90a
Model value	2.917	0.057	0.902	0.941	0.913	0.932

*RMSEA, root mean square error of approximation; GFI, goodness of fit index; CFI, comparative fit index; NFI, normed fit index; TLI, non-normed fit index. a, According to Bentler and Bonett (1980) and Lee et al. (2012); b, According to Browne and Cudeck (1989) and Lee et al. (2012).*

### Hypotheses Testing

**FIGURE 2:**
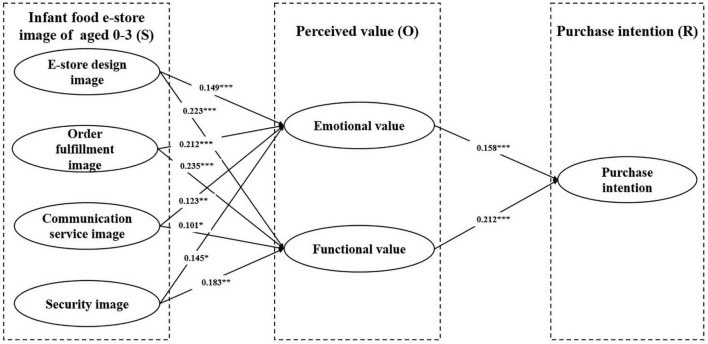
Results of the research model tests. *p < 0.05, **p < 0.01, and ***p < 0.001; n.s., non-significant at the 0.05 level.

After examining the measurement validity and reliability, we tested the proposed hypotheses using AMOS. Table 5 and Figure 2 indicate that 14 of the 15 hypothesized relationships are supported. E-store design image significantly influenced emotional value (H1-1, β = 0.149, *p* < 0.001), significantly influenced functional value (H1-2, β = 0.223, *p* < 0.001). The order fulfilment image significantly influenced emotional value, supporting H2-1 (β = 0.212, *p* < 0.001), significantly influenced functional value, supporting H2-2 (β = 0.235, *p* < 0.001). Communication service image significantly influenced emotional value, supporting H3-1 (β = 0.123, *p* < 0.01), significantly influenced functional value, supporting H3-2 (β = 0.101, *p* < 0.05). The security image significantly influenced emotional value (H4-1, β = 0.145, *p* < 0.05), significantly influenced functional value (H4-2, β = 0.183, *p* < 0.01). Emotional value (β = 0.158, *p* < 0.001), and functional value (β = 0.212, *p* < 0.001) positively influenced purchase intention, thereby supporting H5, and H6.

**TABLE 5:** Results of hypotheses testing.

Research hypothesis	Path value	S.E.	*t*-value	*p*-value	Support
H1-1: E-store design image → Emotional value	0.149	0.040	3.712	***	Yes
H1-2: E-store design image → Functional value	0.223	0.041	5.501	***	Yes
H2-1: Order fulfillment image → Emotional value	0.212	0.045	4.674	***	Yes
H2-2: Order fulfillment image → Functional value	0.235	0.045	5.202	***	Yes
H3-1: Communication service image → Emotional value	0.123	0.046	2.677	0.007**	Yes
H3-2: Communication service image → Functional value	0.101	0.045	2.252	0.024*	Yes
H4-1: Security image → Emotional value	0.145	0.065	2.250	0.024*	Yes
H4-2: Security image → Functional value	0.183	0.064	2.859	0.004**	Yes
H5: Emotional value → Purchase intention	0.158	0.038	4.212	***	Yes
H6: Functional value → Purchase intention	0.212	0.039	5.388	***	Yes

*<0.05; **<0.01; and ***<0.001. n.s., not significant.

### Mediating Effect Testing

Emotional value and functional value mediate the effect of e-store design image, order fulfillment image, communication service image, and security image on consumers’ purchase intention, and the bootstrapping approach was used to test this mediating effect (Preacher and Hayes, 2008). Use and testing of the mediating effects are the main trends in management studies. In SEM, the conceptual model of this study belongs to the multiple mediator model, with two mediating variables (emotional and functional). The analysis of SEM based on bootstrapping can overcome the shortcomings of traditional testing methods, such as the Sobel test in dealing with small sample sizes and small mediating effect values and can bring multiple mediating variables into the model at the same time to gain a deeper understanding of complex management phenomena (Cheung and Lau, 2008). This method can estimate the mediation effect more accurately when a multiple mediator model is used (Muthén, 2010). Table 6 shows that the mediating effect of emotional and functional value on the relationship among e-store design image, order fulfillment image, communication service image, security image, and consumers’ purchase intention are significant with a 95% bootstrap confidence interval, excluding zero. This finding suggests that emotional value and functional value mediate the effect of e-store design image, order fulfillment image, communication service image, and security image on consumers’ purchase intention.

**TABLE 6:** Results of mediating effect analysis.

Constructs	Mediator variables	Effect	SE	CIs	*p*-value	Hypothesis
E-store design image	Emotional value	0.060	0.014	[0.035, 0.092]	0.001**	H7-1
	Functional value	0.089	0.018	[0.058, 0.129]	0.001**	H8-1
Order fulfilment image	Emotional value	0.077	0.017	[0.047, 0.113]	0.001**	H7-2
	Functional value	0.094	0.019	[0.061, 0.136]	0.001**	H8-2
Communication service image	Emotional value	0.056	0.016	[0.029, 0.092]	0.001**	H7-3
	Functional value	0.060	0.018	[0.030, 0.099]	0.001**	H8-3
Security image	Emotional value	0.071	0.021	[0.035, 0.120]	0.001**	H7-4
	Functional value	0.090	0.024	[0.049, 0.144]	0.001**	H8-4
95% Bootstrap confidence intervals for indirect effect						

**p < 0.01.

## Discussion and Implications

### Discussion of Findings

The results show that the 14 hypotheses in this study are all supported. First, the e-store design image, order fulfillment image, communication service image, and security image have a significant positive effect on emotional value and functional value. This shows that baby food e-store image (for ages 0–3) has different effects on consumers’ perceived value. The research conclusion shows that improving online store image is an effective way to improve the perceived value of online consumers.

Second, consumers’ perceived value of emotional value and functional value has a significant positive effect on consumers’ purchase intention. According to the “Customer Value” theory, consumers’ purchase intention depends on their perceived value. Therefore, this study believes that improving the functional value and emotional value of baby food e-stores is also an effective way to improve consumers’ purchase intentions.

Third, perceived value can have a significant mediating impact on the purchase intention of consumers of baby food e-stores. According to the “S-O-R” theory, consumers are “stimulated” by the image of baby food online stores for ages 0–3, and the “response” of purchase intention is generated through the mediating role of perceived value. The results show that baby food e-store image not only has a significant positive impact on perceived value but also on purchase intention.

### Theoretical Implications

In terms of academic research contribution, this study successfully confirmed the suitability of the S-O-R model in explaining the influence of baby food e-store image on perceived value and consumer purchase intention. First, few studies have empirically examined baby food e-stores for ages 0–3, which makes this segment of the market a new research field. This study explores the role of perceived values in influencing customers’ purchase intention in baby food e-stores based on the S-O-R framework, which enriches the research in the field of baby food e-store image and provides a new direction for future research.

Second, few studies have established the link between baby food e-store image (for ages 0–3) and consumer perceived value perspective. This study introduces the theory of perceived value into the field of baby food e-stores and proposes the importance of perceived value. Moreover, a theoretical model is constructed with the consumer perceived value as the mediator variable, which is helpful for future research on the perception of baby food e-stores (for ages 0–3).

Third, this study was carried out based on the S-O-R model, and the findings indicate that e-store design image, order fulfillment image, communication service image, and security image are indirect predictors of consumers’ purchase intention. On the premise of summarizing the image of the baby food e-store and drawing lessons from previous research, multi-dimensionally studied the influencing factors of purchase intention. The establishment and verification of this research model can provide a reference for future research. Therefore, this study expanded the application field of the S-O-R model and improved the body of knowledge about perceived value.

### Practical Implications

This study offers useful managerial implications from three perspectives. First, we comprehensively improve the overall image of baby food e-stores (for ages 0–3) and enhance the competitive advantage of online stores. Today’s Internet shopping market is booming, bringing unlimited vitality to the entire national economy and social development. This study found that baby food e-store image has a positive impact on perceived value, and perceived value has a positive impact on online consumers’ purchase intention. Therefore, to enhance their competitive advantage, baby food e-stores must be committed to improving the overall image of online stores and establishing a distinctive online store.

Second, we implement targeted business service plans to meet the online shopping needs of online consumers. From the perspective of realizing and satisfying consumers’ purchasing intention, baby food e-stores need to grasp the psychological and behavioral characteristics of different consumers. To ensure the quality of the products or services, they should provide superior products. Furthermore, they should attach importance to the image of communication services and provide quality customer service to online consumers. In addition, they should pay attention to the image of order fulfillment, fulfillment of promises, and operate honestly.

Third, this study enhances the value perception of online consumers and implements a humanized business philosophy. Baby food e-stores must first provide products or services that meet the actual needs of consumers and grasp the actual needs of consumers. In the process of communicating with online consumers, online shops should pay attention to emotional communication with consumers, and they should fully understand the actual thoughts, psychological intentions, and behavioral intentions of online consumers.

### Limitations and Future Research

The limitations of this study should be considered before generalizing the findings. First, this study was conducted with data collected from a baby food e-store (for ages 0–3) in China. The results of this study might be different if the model was retested in a different context or a different cultural environment. In the future, scholars should further test and validate our findings in different contexts and cultural environments. Second, this study considered four dimensions of baby food e-store image as independent variables. It is necessary to identify and determine, at a meticulous level, other dimensions manifested like e-store design image, order fulfillment image, communication service image, and security image. Future research could replicate this study using experiments to control for other baby food e-store image dimensions. Finally, given the scope of this study, other variables were not included in the model. Future studies could extend our model to include different consumer psychological variables as mediators or moderators, including consumers’ goals and needs, achievement motivation, and baby food e-store activity involvement.

## Data Availability Statement

The raw data supporting the conclusions of this article will be made available by the authors, without undue reservation.

## Ethics Statement

The studies involving human participants were reviewed and approved by the Normal School, Changshu Institute of Technology. Written informed consent for participation was not required for this study in accordance with the national legislation and the institutional requirements.

## Author Contributions

CJ designed the study and drafted the initial manuscript. XS and LW collected the data, performed the statistical analysis, and drafted the initial manuscript. XS contributed to the revised manuscript. All authors discussed the results and contributed to the final manuscript.

## Conflict of Interest

The authors declare that the research was conducted in the absence of any commercial or financial relationships that could be construed as a potential conflict of interest.

## Publisher’s Note

All claims expressed in this article are solely those of the authors and do not necessarily represent those of their affiliated organizations, or those of the publisher, the editors and the reviewers. Any product that may be evaluated in this article, or claim that may be made by its manufacturer, is not guaranteed or endorsed by the publisher.

## Appendix A

**Table:**

Construct & Item
E-store design image	1. The design of the online store left a good impression on me.
	2. The online store has detailed product introduction, picture displays, and purchase process.
	3. The content news of the online store is updated from time to time.
Order fulfillment image	1. The online store promises to deliver goods in a short time.
	2. The online store offered “freight insurance” for customers.
	3. The goods are received intact without damage.
Communication service image	1. The customer service staff of the online store are warm and polite.
	2. The customer service staff of the online store have sufficient professional knowledge.
	3. The online store will consider accepting customers’ suggestions.
	4. The online store will show concern to me in the form of SMS, telephone, e-mail, etc.,
Security image	1. My personal information is confidential in this online store.
	2. Credit card and payment media information is secure.
	3. The online store has a good reputation and can guarantee goods.
Emotional value	1. I feel happy when purchasing goods in this online store.
	2. I like to purchase the goods of the online store.
	3. The goods in this online store can meet my spiritual needs.
Functional value	1. I can choose the right goods in this online store.
	2. The quality of the goods in this online store can be trusted.
	3. The goods or services of the online store have practical value.
	4. The quality of the goods in this online store is up to standard.
Purchase intention	1. I am more likely to buy in this online store than other stores.
	2. I will continue to buy goods in this online store.
	3. I would recommend the goods and services of the online store to my relatives and friends.
